# Role of Chronic Lymphocytic Leukemia (CLL)-Derived Exosomes in Tumor Progression and Survival

**DOI:** 10.3390/ph13090244

**Published:** 2020-09-14

**Authors:** Nancy Nisticò, Domenico Maisano, Enrico Iaccino, Eleonora Vecchio, Giuseppe Fiume, Salvatore Rotundo, Ileana Quinto, Selena Mimmi

**Affiliations:** 1 Department of Experimental and Clinical Medicine – University Magna Graecia of Catanzaro, 88100 Catanzaro, Italy; nancynistico@unicz.it (N.N.); maisano@unicz.it (D.M.); eleonoravecchio@unicz.it (E.V.); fiume@unicz.it (G.F.); quinto@unicz.it (I.Q.); 2 Department of Health Sciences–University Magna Graecia of Catanzaro, 88100 Catanzaro, Italy; srotundo91@gmail.com

**Keywords:** CLL, exosomes, miRNAs

## Abstract

Chronic lymphocytic leukemia (CLL) is a B-lymphoproliferative disease, which consists of the abnormal proliferation of CD19/CD5/CD20/CD23 positive lymphocytes in blood and lymphoid organs, such as bone marrow, lymph nodes and spleen. The neoplastic transformation and expansion of tumor B cells are commonly recognized as antigen-driven processes, mediated by the interaction of antigens with the B cell receptor (BCR) expressed on the surface of B-lymphocytes. The survival and progression of CLL cells largely depend on the direct interaction of CLL cells with receptors of accessory cells of tumor microenvironment. Recently, much interest has been focused on the role of tumor release of small extracellular vesicles (EVs), named exosomes, which incorporate a wide range of biologically active molecules, particularly microRNAs and proteins, which sustain the tumor growth. Here, we will review the role of CLL-derived exosomes as diagnostic and prognostic biomarkers of the disease.

## 1. Introduction

Chronic lymphocytic leukemia (CLL) represents a very heterogeneous type of adult leukemia, with a variable course of the disease. In fact, some patients show no symptoms and remain stable for many years without therapy, while others develop early symptoms with rapid disease progression requiring therapy [1]. To date, several molecular biomarkers have been associated with CLL prognosis, which include immunoglobulin heavy-chain variable region gene (IGHV) mutation status, chromosomes abnormalities, proteins or miRNA expression levels, genetic mutations or epigenetic modifications [2]. The biochemical and clinical evaluations of patients are both required for monitoring the disease remission after therapy.

CLL cells have a high rate of proliferation and tumor progression in vivo into the germinal centers [3]. However, several studies have demonstrated the inability of CLL cells to proliferate in vitro or ex vivo after their isolation from peripheral blood [4,5]. This evidence suggests the requirement of CLL cells interaction with the tumor microenvironment in vivo for maintaining tumor cells proliferation and survival [6,7]. Indeed, the tumor growth in vivo is influenced by the tumor microenvironment, including fibroblasts, immune system cells, vascular and lymphatic network cells, as well as the extracellular matrix components, such as cytokines and stroma components [8,9]. The involvement of tumor microenvironment and BCR stimulation for CLL growth have been confirmed by a recent study, describing the optimal in vitro culture conditions of CLL cells mimicking the in vivo compartments [10].

In the last decades, many research groups focused their attention on the so-called nurse-like cells (NLCs), mononuclear peripheral blood-derived cells found in the germinal centers, which promote the survival and proliferation of CLL cells [11,12,13]. The communication between CLL and NLCs requires a very closed interaction because it involves specific receptors binding and cytokine relapse in the extracellular space [14].

An additional “remote” communication of CLL cells has been recently identified. This mechanism involves novel molecular structures, named exosomes, which are 30–100 nm diameter extracellular vesicles (EVs) secreted by all cell type [15,16]. When discovered, the exosomes were considered a sort of “rubbish bin” used by the cells to remove unuseful materials. Then, the role of exosomes has been progressively updated as functional delivery vesicles from parental cells to other tissues, emerging as potential biomarkers of diseases progression in human as well as in animals [17]. Indeed, they are now considered new and important actors in cellular cross-talk at closed and long distance, thus enriching the modality of cellular communications, which were initially a prerogative of hormones and cytokines [18,19].

The exosomes carry functionally active biological molecules, such as proteins, lipids, messenger RNA (mRNA) and microRNA (miRNA), stored by the parental cells during the exocytosis [20]. Via this process, the exosomes deliver their cargo across bloodstream and lymphatic vessels and pour their content into the target cells, influencing their metabolism and phenotype [21].

Several studies have demonstrated that the total amount of exosomes is much higher in pathological conditions respect to healthy controls, as reviewed by Yu-Ling Tai et al. [22]. However, it is relatively unknown whether the content of exosomes may differ in pathological conditions. Indeed, the number of exosomes as well as the cargo composition may differ in normal and tumor conditions, reflecting the tumor heterogeneity [23]. Circulating miRNAs have been extensively described as potential biomarkers of several diseases [24,25,26,27].

In this review, we aim to summarize the role of miRNAs delivered by CLL-derived exosomes and highlight their potential role as “red flag” of minimal residual disease and poor outcome of the disease. We will also discuss the function of exosomal proteins as potential signaling molecules in the crosstalk between CLL cells and tumor microenvironment.

## 2. Exosomes: The Low-Cost Carriers

First identified in the 1980s [28,29], exosomes, or more generally the EVs, represent nowadays the fastest way of cell–cell communication in neoplastic diseases, mediating tumor progression and metastatic niches establishment [30]. The relapse of exosomes involves the endosome machinery through invaginations of plasma membranes [31]. Endosome maturation leads to the formation of multivesicular bodies (MVBs) containing the exosomes, which are named intraluminal vesicles (ILVs) at this stage. The formation and relapse of exosomes occur through the endosomal sorting complexes required for transport (ESCRT)-dependent and ESCRT-independent mechanisms [32].

In the ESCRT-dependent mechanism, the generation of exosomes requires the ESCRT complex composed by four proteins (from ESCRT-0 to ESCRT-3), which cooperate with several accessory proteins. This process begins when ESCRT-0 binds and clusters ubiquitinated proteins on cell surface. This complex contacts ESCRT-1 and ESCRT-2 for the generation of multivesicular body (MVBs). Then, ESCRT-3 is responsible of the formation of the constriction zone. The energy for stripping the complex from the cellular membrane at the end of each step is provided by the Vps4-Vta1 proteins, equipped with ATPase activity. The removal of ubiquitin tags from proteins initially contacted by ESCRT-0 occurs trough the recruitment of Bro1 protein associating the deubiquitinase Doa4 [33]. Then, the TSG101 and CHMP4 proteins operate the endosomal membrane budding and abscission [34].

The ESCRT-independent mechanism involves the ceramide molecules that belong to the sphingomyelin family. Several data demonstrate the role of ceramide in the generation of MVBs by using inhibitors, such as siRNA for nSMase2 or the ceramide inhibitor GW4869, which reduce the exosomes relapse [35]. Interesting, exosomes released through ceramide-depended process are enriched in proteolipid protein (PLP) compared to ESCRT-dependent process exosomes [36]. In these processes the membrane structures envelope the cytosolic components representing the functional cargo of exosomes. Following specific input such as BCR stimulation, the MVBs fuse with plasma membranes and the exosomes are released in the extracellular compartment in order to move in the microenvironment in search of the target cells [37], in which they can modulate different downstream processes [38,39]. Otherwise, exosomes are transferred to lysosome compartment for degradation [40].

The composition of exosomal cargo depends on the parental cell with a high heterogeneity among all circulating exosomes. Exosomes can transport nucleic acids, (e.g., DNA, mRNA, miRNA), proteins (e.g., tetraspanins, heat shock proteins), lipids (e.g., cholesterol, sphingomyelin, ceramide), and Fas ligands [41,42,43]. Several proteins, such as CD63, CD81, CD9, TSG-101, Syndecan-1, MHC molecules, ALIX, HSP70 and BCR, are located on the surface of exosomes or included as cargo of exosomes and may represent markers of the paternal cell [44,45,46,47,48]. The composition and quantity of cargo components can differ between the normal and disease- associated exosomes [49,50,51]. This evidence suggests that the exosomes derived from pathological cells may be involved in pathogenesis of the disease and be considered a potential diagnostic and/or prognostic tool.

In the context of B cell malignancies, CLL-derived exosomes show the phenotypic characteristics of normal exosomes, such as CD63 and CD81 expression, but they additionally express IgM, CD19 and Lyn; this could be used to distinguish the neoplastic B cells-derived exosomes among the assorted groups of circulating EVs [52]. The release of exosomes does not represent a constitutive mechanism in B cells, but it is induced by external stimulations supported by several signaling interactors such as IL-4, anti-CD-40, or BCR [53,54,55]. In particular, IL-4 can be considered a common factor for the release of exosomes in different cell types as it has been found to cause the release of exosomes into mast cells [56]. In this contest, inhibitors of these stimulator factors could be exploited to reduce or block the release of exosomes by neoplastic B cells in order to prevent the communication with the tumor microenvironment and the resulting tumor progression.

**Figure 1:**
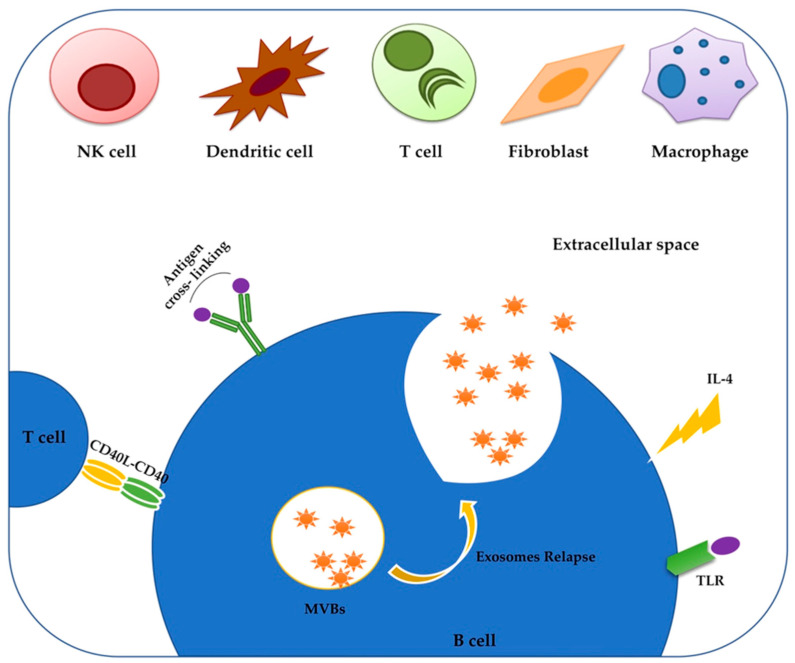
Exosomes relapse from a primary B cell after stimulation. Both ESCRT-dependent and ESCRT-independent mechanisms of exosomes relapse, require exogenous stimuli on several receptors on B-cell membrane (CD4, BCR, TLR, IL-4R) to start the production and secretion of exosomes. This induces the fusion of the multivesicular bodies (MVBs), containing the exosomes, with the plasma membrane. Via this process, the exosomes containing cytosolic components of parental cell are released into the extracellular compartment and move toward target cells, in which they can modulate different downstream processes.

In Figure 1, we represent a B cell releasing exosomes in response to external stimuli, such as BCR triggering by antigen or CD40-CD40L binding.

Once produced and released in blood vessels or interstitial fluids, CLL-derived exosomes can contact and modulate several accessory cells in the tumor microenvironment, in order to support the proliferation and progression of tumor cells by inducing or suppressing a wide range of molecular events [57,58,59]. A well-known effect of CLL-derived exosomes is the transformation of stromal cells into cancer-associated fibroblasts, which promotes cytokine production, inflammation and angiogenesis, thus sustaining tumor survival and progression [57]. Monocytes and macrophages in the tumor microenvironment may protect tumor cells by assuming a pro-tumorigenic phenotype with the expression of several immunosuppressive proteins, such as the programmed cell death 1 ligand 1 (PD-L1). This phenotype remodulation is mediated by CLL-derived exosomes that pour their content in the target monocytes [58,59].

Thus, CLL cells switch the tumor microenvironment by exosomes relapse in order to create a sort of “glass niche”, where tumor B cells can proliferate and survive. On the other side, bone marrow mesenchymal stromal cells (BM-MSCs) are able to produce EVs that modulate CLL cells [60]. Indeed, it has been reported the increase of BM-MSCs–derived exosomes in CLL patients respect to healthy donors with the evidence of a strict correlation between the CLL uptake of BM-MSCs-derived exosomes and their migration capacity toward proliferation niches associated with apoptosis resistance [60].

## 3. miRNAs of CLL-Derived Exosomes Modify the Tumor Microenvironment

Among the wide range of molecules delivered by exosomes, we will focus on miRNAs produced by CLL cells. miRNAs are small functional non-coding RNA molecules (~20 nucleotides) that influence the protein expression by post-transcriptional silencing through direct transcript degradation or translational inhibition [61]. The expression of miRNA is not ubiquitarian as it depends on the cell of origin and physiological/pathological conditions [61]. Two processes of miRNAs biogenesis are known: the canonical pathway, which is the most used by cells, and the non-canonical pathway [62]. Once produced, miRNAs can modulate gene expression by the binding to the 3′-untranslated region (3′-UTR) of mature mRNA targets, inducing mRNA degradation or translational suppression [63].

miRNAs have attracted a large interest in the scientific community as they are useful for RNA interference (RNAi) of mammalian genes in tissue culture in order to address the function of target genes [64,65]. The capability of miRNAs to modulate the gene expression plays an important role in tumorigenesis. Tumor cells and tumor microenvironment modulate the expression levels of miRNAs for increasing the expression of pro-survival genes and repressing the anti-tumorigenic genes. For example, the transacting factor Myc is overexpressed in cancer and this event is associated with the overexpression of a set of miRNAs, which inhibit their target tumor suppressor genes [66]. Furthermore, miRNAs can act as tumor promoters, assuming the alias of “oncomiRs”, when they silence pro-apoptotic or anti-proliferative genes [67].

miRNAs were first identified as plasma circulating molecules influencing pathogenesis and survival of leukemia cells [68,69]. Several data have recently reported the delivery of miRNAs in CLL-derived exosomes. Table 1 summarizes the most abundant miRNAs found in the CLL-derived exosomes and their functions.

CLL cells select the miRNAs to encapsulate into the circulating exosomes in order to modulate the expression of their target gene in favor of tumor proliferation and progression.

Exosomes released by CLL cells are enriched in miR-202-3p. The target gene of miR-202-3p is the ‘suppressor of fused’ (Sufu) increase, a Gli-Hedgehog signaling pathway modulator implicated in pattern formation and cellular proliferation during development. The reduction of intracellular level of this miRNA results in the increase of the expression level of Sufu, supporting survival and progression of tumor cells [52,70].

miR-146a and miR-451 are other two miRNAs shuttled by CLL-derived exosomes more than normal B cells exosomes; they induce the transition of stromal cells toward cancer-associated fibroblasts (CAFs) [57]. Indeed, the CLL-derived exosomes carrying miR-146a and miR-451 contact stromal cells in which they release their cargo and induce the activation by phosphorylation of several kinases. As result, activation of RAC-alpha serine/threonine-protein kinase (AKT), extracellular signal-regulated kinase (ERK)1/2, C-AMP response element-binding protein (CREB) and glycogen synthase kinase (GSK)3a/b promote an inflammatory phenotype with the reorganization of the cells toward the CAFs phenotype. Then, CAFs secrete inflammatory molecules, such as cytokines and proangiogenic factors, and thus promote the tumor progression and metastasis [71,72].

miRNAs expression levels (intracellular or serum levels) are often associated with the common phenotypic features of aggressive CLL. Among them, miR-150 plays a critical role in the hematopoiesis process, especially in the differentiation and development of lymphoid lineage [73]. Analyzing also the common molecular features of poor prognosis, miR-150 results associated with the expression of the ζ-chain-associated protein of 70 kDa (ZAP-70) or the unmutated immunoglobulin heavy chain variable (IGHV) genes [74]. Despite the large amount of circulating miR-150, free or associated with serum proteins, the CLL-derived exosomes can assemble miR-150 in order to protect it from the RNAse and thus sustain its pro-tumorigenic action [75].

As previously mentioned, CLL is a very heterogenous B cell malignancy with a wide range of behaviors from patient to patient. Since 1928, a very aggressive and invasive form of lymphoma derived from CLL, named Richter’s syndrome (RS) has been described [76,77,78,79]. A recent work has analyzed the circulating EVs in both CLL and RS patients and discovered high levels of miR-19b as predictor of the evolution of drug-resistant CLL toward RS, resulting in down-regulation of TP53 and up-regulation of MKI67, as mechanism of tumor cell proliferation, survival and invasion [80]. 

miR-155 family is highly expressed in both mature B- and T-cells, playing a key role in hematopoietic lineage differentiation, immunity regulation, inflammation, viral infections and also cancer [81]. Analysis of CLL-derived exosomes revealed an abundant amount of miR-155, with the function to induce and hold the myeloid-derived suppressor cells (MDSCs) in a vitamin D-dependent process [82].

By quantitative RT-PCR, Yeh et al. [55] investigated the whole set of miRNAs, which could be possibly deregulated in both CLL cells and CLL-derived exosomes compared to the normal counterpart. They demonstrated that miR-29 was equally expressed in normal B cells and CLL cells, while CLL-derived exosomes showed higher levels of miR-29 respect to normal exosomes [55]. miR-29 seems to play a key role in hematological malignancies by regulation of TCL1, MCL1 and DNA-methyltransferases [83]. In the same study, miR-223 was downregulated in CLL-derived exosomes respect to the healthy controls [55]. As putative prognostic factor, miR-223 was downregulated in aggressive stages of CLL and was associated with poor prognostic factors [84].

MEC-1, a well-established CLL cell line, also secrete exosomes in cell culture in vitro [52]. MEC-1 derived exosomes are enriched of Y RNA, especially the hY4 full length and the 31nt-fragment, as well as in the exosomes secreted by breast cancer cells and glioma cells [85,86,87]. hY4 enriched exosomes are captured and internalized by monocytes in tumor microenvironment, inducing the release of several chemokines, inflammatory interleukins and immunosuppressive factors [87].

As additional class of non-coding RNAs, the circular RNAs (circRNAs) also play a crucial role in tumor pathogenesis and progression. They can be nuclear genome-derived (nu)-circRNAs and mitochondrial genome-derived (mt)-circRNAs [88,89]. In the CLL context, a recent study demonstrated that CLL-derived exosomes are enriched of mt-circRNAs, with upregulation of mt-COX2 expression [90]. Since the overexpression of mt-COX2 seems to be associated with leukemogenesis and a poor prognosis, this observation lays the foundations for the endorsement of mt-COX2 as new prognostic marker of CLL.

## 4. Proteins Delivered by CLL Exosomes Modulate Intracellular Pathways in Favor of CLL Progression

Proteins are another class of bioactive molecules shuttled by normal or tumor-associated exosomes. A deep comprehension in terms of proteomic differences among pathological and normal exosomes can be exploited in search of novel biomarkers for diagnosis and prognosis of hematological diseases, such as CLL. To date, there is a lack of information on the proteomic cargo of CLL-derived exosomes.

Few studies have investigated the proteomics of exosome cargo. Recently, it was demonstrated that the proteins contained in CLL exosomes exhibit a recurrence in a repeated motif in the amino acid sequence. In fact, analyzes conducted by mass spectrometry have demonstrated the presence of proteins showing the KFERQ sequence (Lys–Phe–Glu–Arg–Gln) in 67% of the CLL exosome proteome in comparison with the cell proteome [57]. The current hypothesis about the role of that specific motif is about the targeting of proteins to the multivesicular bodies, opening a major point on the existence of a specific sorting mechanism, almost yet to be exploited.

Two proteins carried by CLL-derived exosomes involved into deregulation of cell cycle pro-proliferation and into immune-escape have been identified: S100 calcium-binding protein A9 (S100-A9) and BCL2-associated athanogene 6 (BAG-6).

S100-A9 is normally involved in the regulation of cell cycle progression and cell differentiation. In B cell disorders, it is implicated in the abnormal differentiation of myeloid cells in the tumor stroma and leukemia progression [91]. Plasma-derived exosomes from patients with progressive CLL exhibit high levels of S100-A9 protein compared to indolent CLL patients. The increased level of S100-A9 is associated with hyper-activation of nuclear factor kappa-light-chain-enhancer of activated B cell (NF-κB), inducing the phosphorylation coupled to proteasomal degradation of the inhibitor IκB-α [92].

The sidestepping of the immune system represents the first stage of tumor progression, especially by the inhibition of natural killer (NK) cells. NK cells are considered the best anti-tumor immune response player and the deregulation of their activity is reported in several malignancies, including CLL [93,94]. Soluble human BCL2-associated athanogene 6 (BAG-6) is the natural ligand of NKp30 receptor for NK activation. In CLL patients’ soluble serum BAG-6 is detectable at the progressive stage of the disease, meanwhile, NK are only activated by BAG-6 expressed by exosomes. The deregulation of the ratio soluble/exosomal-BAG-6 CLL plasma serum promotes tumor escape of immune-surveillance and survival of neoplastic B cells [95].

## 5. Concluding Remarks

CLL is a lymphoproliferative disease with a variable clinical course [96]. Some CLL patients remain stable for years without requiring any drug treatment and thus they can conduct a quasi-ordinary life. Unfortunately, some other patients experiment an aggressive disease that requires therapy. These patients can develop therapy resistance with minimal residual disease with poor prognosis and fatal outcome [97,98,99].

Canonical parameters for CLL diagnosis and prognosis are based on lymphocytes count (total white blood cells) and their phenotypic analysis by flow cytometry using standard antibodies, such as anti-CD19, anti-CD20, anti-CD5 and others [100]. Aggressive behavior of the disease may be predictable by evaluating the ζ-chain-associated protein of 70 kDa (ZAP-70) or CD38 expression on CLL surface membrane by flow cytometry [101]. In some cases, these parameters have been questioned, and other biomarkers of aggressiveness have been proposed, such as the inhibitor of Bruton’s tyrosine kinase (IBTK), a well-characterized pro-survival protein [102,103,104,105,106]. The mutational status of heavy chain variable region of BCR is also considered a predictor biomarker of the aggressiveness of the disease. In particular, the unmutated status, corresponding to a mutation rate less than 2% compared to the germline sequence, reflects a poor response to therapy with disease recurrence and poor prognosis [107,108]. As diagnostic tool, the sequence analysis of the immunoglobulin receptor is a long time procedure, which often involves external service laboratories, thereby increasing the costs and the time of diagnosis. The clinical evaluation of lymph nodes involvement, spleen and liver volume is essentially required for diagnosis and prognosis [100]. All these investigations are often “touchable” in a late stage of the disease and poor prognosis is foregone. So, the scientific research goal would be looking for novel and timesaving reliable markers for early diagnosis and prognosis of the disease, when all the parameters mentioned above are still not touchable.

In the minimal residual disease, the 5T33MM-derived exosomes were earlier detected in vivo by specific Id-peptides targeting, compared to the conventional paraprotein in a mouse model of multiple myeloma (MM) engrafted by 5T33MM murine cell line [48]. This evidence suggests that tumor-derived exosomes could be a useful tool similar to a magnifying glass for detecting tumor progression at early stage, in order to promptly start the treatment and prevent a worsening of the disease. Furthermore, the use of small Id-peptides as specific targeting tools provides a novel and innovative methods to discriminate tumor-derived exosomes among the total amount of exosomes released in the patient.

Given the encouraging results of the MM murine model, it could be possible to translate the same approach to other hematological diseases, including CLL. Indeed, CLL-derived exosomes could be detected and analyzed as early biomarkers of disease recurrence, when flow cytometry assay is not adequately suited to detect the neoplastic cells at the first step of expansion because they are still enclosed into the germinal centers. Exploiting the versatility of cell-specific small peptides to be functionalized with fluorophores or biotin [109,110,111,112] and the feasibility of liquid biopsy methodology in contributing to diagnosis and prognosis profiling of cancer [113,114,115], it could be possible to perform an innovative and rapid diagnostic/prognostic tool, of course without putting aside the gold standards actually used in clinical practice. Furthermore, according to the evidence that in the same patient more than one neoplastic B cell clone of CLL could co-exist [97,98], we should find more than one CLL-derived exosomes group, each one with different cargo reflecting the paternal B cell clone.

It has been reported that patients affected by CLL show a higher amount of circulating exosomes in serum respect to healthy conditions [30]. The deeper analysis of the molecules expressed on the exosomes surface or shuttled by these circulating vesicles could give more information in term of aggressiveness and clinical outcome prediction. To this end, it would be necessary to perform a laboratory protocol able to distinguish tumor-derived exosomes among the variegate range of circulating EVs [48,116]. Then, it would be useful to distinguish the single groups of CLL-derived exosomes in that patients harboring two or more tumor B cell clones. All these data could be exploited to cluster CLL patients based on phenotypic features of exosomes. In this regard, it is worthwhile to remind that CLL subpopulations can be distinguished by the IgBCR expression, which is specific of each CLL clone [97]. Further, CLL clones can be monitored with peptide ligands that are specific of each CLL sub-population [98]. As the IgBCR is also expressed in exosomes derived from CLL clones, in principle peptide ligands of the parental CLL clone could also detect derivative tumor exosomes, thus allowing their selection and molecular analysis as well as the disease monitoring.

Several data demonstrate that specific genes are deregulated in intracellular pathways involved in B cell tumorigenesis and can be targeted with tumor- and patient-specific therapy [110,117,118]. By analyzing the deregulated CLL exosomal proteins or miRNAs in the blood of patients we could have reliable tool for rapid diagnosis of disease recurrence and selection of the most appropriate tumor-targeted therapy. If as first instance these small vesicles have attracted the attention of the scientific world for their role in the early diagnosis and prognosis overall in disease recurrence, recently the efforts of part of the scientific community are now focusing also on their therapeutic potential [119,120]. Thanks to their molecular structure mimicking, the plasma membrane of the cells and their capability to reverse their cargo into target cells, exosomes could be shaped and filled of drug molecules, acting as drug-delivery systems [121].

## Figures and Tables

**Table 1:** Summary of major miRNAs delivered by chronic lymphocytic leukemia (CLL)-derived exosomes. The table shows for each miRNA the expression levels in CLL-derived exosomes respect to normal circulating exosomes (up ↑ or down ↓) and the target gene or modulated process. The resulted expression levels of target genes are inversely proportional to expression levels of relative miRNA.

miRNA	Expression Level in CLL-Derived Exosomes	Target	Reference
miR-202-3p	↑	Sufu gene	Hegde G.V. et al., MCR 2008 [70]
miR-146a/miR-451	↑	Kinases in Stromal cells	Paggetti J. et al., Blood 2015 [57]
miR-150	↑	Hematopoiesis	Stamatopoulos B. et al., Mol Med 2015 [75]
miR-19b	↑	TP53 and MKI67	Jurj A. et al., Crit Rev Clin Lab Sci. 2018 [80]
miR-155	↑	MDSCs induction	Bruns H. et al., Leukemia 2017 [82]
miR-29	↑	TCL1	Yeh YY. et al., Blood 2015 [55]
miR-223	↓	HSP90B1	Yeh YY. et al., Blood 2015 [55]
Y RNA	↑	Proliferation	Haderk F. et al., Sci Immunol. 2017 [87]
